# Supplementary material for: Optogenetic Targeting of Astrocytes Restores Slow Brain Rhythm Function and Slows Alzheimer’s Disease Pathology
Source: Res Sq. 2023 Apr 25:rs.3.rs-2813056. Preprint. [Version 1] doi: 10.21203/rs.3.rs-2813056/v1 (PMC10168443; doi:10.21203/rs.3.rs-2813056/v1)
Supplement: Supplement 1 [file NIHPPrs2813056v1-supplement-1.pdf]

Supplementary Files

This is a list of supplementary files associated with this preprint. Click to download.

- AstrocyteMSScientificReportssupplementsubmittted.docx
